# Nitrogen and boron nutrition in grafted watermelon I: Impact on pomological attributes, yield and fruit quality

**DOI:** 10.1371/journal.pone.0252396

**Published:** 2021-05-28

**Authors:** Kemal Yalçın Gülüt

**Affiliations:** Department of Soil Science and Plant Nutrition, Faculty of Agriculture, Çukurova University, Adana, Turkey; Harran Üniversitesi: Harran Universitesi, TURKEY

## Abstract

Watermelon is extensively consumed fruit across the globe. However, limited is known about interactive effect of nitrogen (N) and boron (B) nutrition on pomological, yield and fruit quality attributes of grafted watermelon. This two-year study tested the influence of different N and B doses on pomological, yield and fruit quality attributes of grafted watermelon under field conditions in Çukurova plains of Turkey. Four different N (0, 90, 180 and 270 kg ha^-1^) and two B doses (0 and 2 kg ha^-1^ B) were tested. The individual and interactive effects of N and B significantly altered pomological, yield and fruit quality attributes during both years. Overall, application of 270 kg ha^-1^ N and 2 kg ha^-1^ B improved yield, pomological and fruit quality attributes during both years. The highest values for yield, main stem length, stem diameter, fruit weight, fruit width, number nodes and branches per stem were recorded for 270 kg ha^-1^ N during both years. However, rind thickness was not altered by N application. Similarly, the highest values for quality attributes such as sucrose, glucose, fructose, citric acid, tartaric acid and ascorbic acid were noted for 270 kg ha^-1^ N during both years. Interestingly, no N application and 90 kg ha^-1^ N recorded the highest values of maleic acid during both years. The highest values of rind thickness, fruit length, fruit width and fruit weight were noted for 2 kg ha^-1^ B during both years, while B application had no effect on main stem length, main stem diameter, number of nodes and number of branches. Regarding N by B interactions, 180 and 270 kg ha^-1^ N with both B doses observed the highest values for yield, pomological and quality attributes during each year. These results indicate that N has significant contribution towards yield, pomological attributes and fruit quality of grafted watermelon. Therefore, N should be applied at the rate of 270 kg ha^-1^ for better yield, pomological attributes and fruit quality. Nonetheless, where necessary grafted watermelon should be fertilized with 2 kg ha^-1^ B for better fruit quality and pomological attributes.

## Introduction

Nutrients are the most important component of growing environment for plants and influence their growth and yield. Yield and quality of field crops is positively influenced by major macro nutrients, i.e., nitrogen (N) [1, 2] or N-phosphorus-potassium [3, 4]. Moreover, N affects several traits influencing biomass production [2]. Nitrogen increases the number and size of leaf cells [3, 5]. Moreover, N-availability influences secondary metabolites, plant growth and differentiation processes [6, 7]. However, excessive use of N has been questioned recently due to huge leaching/volatilization losses and adverse effects on the environment.

Nitrogen is regarded as the growth-limiting nutrient due to its role in plant growth and productivity [8]. It is an integral part of numerous biomolecules, including enzymes, structural proteins, chlorophyll, adenosine triphosphate (ATP) and nucleic acids (DNA and RNA). Dry biomass production, leaf area and photosynthetic capacity are directly affected by N. Optimum N supply is mandatory for healthy plant growth and higher crop yield [9]. Thus, majority of crop varieties are selected and developed under optimum N fertilization.

Reproductive and vegetative growth phases, leaf emergence rate, grain yield and yield components are significantly reduced by N-deficiency [10]. Nonetheless, applied N is not used efficiently. For instance, <40% of applied N is taken up and utilized by barley [11]. Hence, farmers are concerned to increase N-use efficiency (NUE) to obtain higher crop yields. The application of N fertilizer has increased by >8-folds since 1961 [12]. This implies that excessive N application and low NUE could have adverse effects, including soil acidification, agriculture-related pollutions, and negative impacts on soil microbial activity [13, 14]. The harmful effects of N losses from the soil have toxicological implications for ecosystems and living organisms [15–17].

Micronutrients are essential in appropriate concentration for plant growth and development. These nutrients play a significant role in most of the physiological processes like photosynthesis, respiration and various enzymatic reactions. Their deficiency or excess disturbs metabolic activities occurring during different plant development stages. Both deficiency and excess can cause chlorosis, necrosis, stunted growth and mottled leaves etc.

Boron (B) has narrow toxicity and deficiency range. The prime function of B is linked to the formation of cell wall and cell membranes, pollination, pollen germination, cell division, translocation of carbohydrates and metabolism of calcium, indole acetic acid and RNA [18]. Boron is many times concomitant with N, phosphorous, potassium and calcium in plants; hence, required for high yield of crops.

Boron is involved in cell wall structural integration and regulates porosity and tensile strength of the cell wall [19]. The plants require higher B than other trace/micronutrients [20]. However, the excess and deficiency of B negatively affects plant growth [21–24]. Boron is found in the soil within 10–300 mg kg^−1^ range [25].

Watermelon (*Citrullus lanatus* L.) is globally important commercial vegetable fruit. China leads watermelon production of the globe, whereas Turkey follows China in terms of production [26]. Turkey has 10% share in global watermelon production. The production is negatively affected by several factors, including plant nutrients [26, 27]. Nitrogen and B are critical nutrients required for optimum production of watermelon [26–30]. However, limited is known about the interactive effect of N and B on yield, pomological attributes and fruit quality of grafted watermelon in Turkey.

Therefore, this study assessed the impact of different N and B doses on yield, pomological and fruit quality attributes. It was hypothesized that increasing N and B doses would improve yield, pomological and fruit quality attributes.

## Materials and methods

### Studied species and experimental site

Watermelon is considered as a xerophytic tropical fruit. Its cultivation is spread over warm regions [31]. The current study was conducted at experimental fields of Research and Application Center, Çukurova University, Faculty of Agriculture, Department of Soil Science and Plant Nutrition, Turkey during watermelon growing seasons of 2018 and 2019. Grafted watermelon cultivar ‘Starburst’ was used as experimental material. The experiment was laid according to split-plot design with N as main factor and B as sub-factor. All experimental treatments had four replications.

Seedlings were planted keeping 4 m distance between rows, 1.2 m between plants and 6 plants were grown in each replication. The soil was analyzed prior to the initiation of experiments and depending on the results of the soil analysis 25 kg of phosphorus (P_2_O_5_) was applied per hectare at the time of planting.

Four different N doses, i.e., N_0_ (0 kg N ha^-1^), N_1_ (90 kg N ha^-1^), N_2_ (180 kg N ha^-1^) and N_3_ (270 kg N ha^-1^) and two different B doses, i.e., B_0_ (0 kg ha^-1^ B) and B_2_ (2 kg ha^-1^ B) were used in the study. Nitrogen was supplied by using urea and applied in three equal splits (i.e., at sowing, flowering and fruiting). Etidot67-B was used as B source and whole amount of B was applied at the time of sowing.

When plants were 45 days old, pomological characteristics such as main stem length (cm), number of branches (pieces), main stem diameter (mm) were measured with the help of meters and calipers in the field. Depending on the climatic conditions (the first week of July), the harvesting process was carried out by hand picking the fruits by drying the auricles and leeches on the fruit stalk. The yield was computed based on the weight of harvested fruits. The pomological features were realized by taking 3 fruits from each parcel.

### Statistical analysis

The collected data for nutrient uptake were tested for normality by Shapiro-Wilk normality test [32]. The data were normally distributed; therefore, original data were used in statistical analysis. The differences among the years were analyzed by paired t test, which were significant. Therefore, data of both years were analyzed and presented, separately. Two-way analysis of variance (ANOVA) was used to test the significant differences among N and B doses, and their interaction [33]. Least significant difference at 5% probability was used to separate the means where ANOVA indicated significant differences.

## Results

### Growth and yield attributes

The individual and interactive effect of N and B significantly altered various growth and yield attributes with some exceptions during both years (Table 1). Nitrogen doses significantly altered all growth attributes except number of branches during both years. However, the growth attributes were not altered by B doses during both years, except for the only significant effect on number of branches during 2^nd^ year. Regarding N by B interactions, all growth attributes were significantly affected except for the non-significant effect on number of branches during 1^st^ year (Table 1).

**Table 1:** Analysis of variance for different pomological traits of grafted watermelon grown under varying nitrogen and boron levels.

		2018				2019			
Rind thickness									
Source	DF	SS	MS	F value	P value	SS	MS	F value	P value
**Nitrogen (N)**	3	3.49	1.16	0.38	0.767	4.18	1.39	0.54	0.6582
**Boron (B)**	1	24.73	24.73	8.07	0.005	10.56	10.56	4.07	0.0466
**N × B**	3	66.21	22.07	7.20	0.000	19.77	6.59	2.54	0.0615
**Fruit length**									
**Nitrogen (N)**	3	4568.49	1522.83	5.65	0.001	7298.79	2432.93	7.05	0.0003
**Boron (B)**	1	11969.45	11969.45	44.44	0.0001	0.09	0.09	0.00	0.9872
**N × B**	3	678.53	226.18	0.84	0.476	440.52	146.84	0.43	0.7351
**Fruit width**									
**Nitrogen (N)**	3	2649.68	883.23	7.97	0.0001	5077.99	1692.66	13.51	0.0001
**Boron (B)**	1	5527.97	5527.97	49.91	0.0001	816.86	816.86	6.52	0.0124
**N × B**	3	821.94	273.98	2.47	0.069	305.32	101.77	0.81	0.4906
**Dry biomass**									
**Nitrogen (N)**	3	25.17	8.39	4.02	0.0100	22.93	7.64	6.88	0.0003
**Boron (B)**	1	10.18	10.18	4.87	0.0299	1.07	1.07	0.97	0.3285
**N × B**	3	10.28	3.43	1.64	0.1859	2.77	0.92	0.83	0.4804
**Fruit weight**									
**Nitrogen (N)**	3	34.03	11.34	11.40	0.0001	57.67	19.22	16.47	0.0001
**Boron (B)**	1	62.97	62.97	63.28	0.0001	7.09	7.09	6.07	0.0157
**N × B**	3	19.21	6.40	6.43	0.0005	0.69	0.23	0.20	0.8980
**Main stem length**									
**Nitrogen (N)**	3	8938.16	2979.39	6.37	0.0006	2.72	0.91	8.16	0.0001
**Boron (B)**	1	27.39	27.39	0.06	0.8094	0.00	0.00	0.01	0.9218
**N × B**	3	5308.35	1769.45	3.78	0.0133	0.56	0.19	1.69	0.1758
**Main stem diameter**									
**Nitrogen (N)**	3	3.75	1.25	1.68	0.1762	42.60	14.20	6.98	0.0003
**Boron (B)**	1	0.34	0.34	0.46	0.4979	0.06	0.06	0.03	0.8621
**N × B**	3	3.50	1.17	1.57	0.2020	4.36	1.45	0.72	0.5455
**Number of nodes**									
**Nitrogen (N)**	3	100.75	33.58	3.80	0.0131	61.48	20.49	3.00	0.0351
**Boron (B)**	1	9.31	9.31	1.05	0.3078	8.01	8.01	1.17	0.2821
**N × B**	3	162.05	54.02	6.10	0.0008	0.84	0.28	0.04	0.9889
**Number of branches**									
**Nitrogen (N)**	3	1.81	0.60	1.29	0.2823	1.54	0.51	1.01	0.3930
**Boron (B)**	1	0.03	0.03	0.06	0.7994	2.08	2.08	4.08	0.0464
**N × B**	3	0.50	0.17	0.36	0.7826	1.74	0.58	1.14	0.3377
**Yield**									
**Nitrogen (N)**	3	2057324999	685774999	89.95	0.0001	1488760839	496253613	16.74	0.0001
**Boron (B)**	1	87738296	87738296	11.51	0.00	490040644	490040644	16.53	0.00
**N × B**	3	101706641	33902213	4.45	0.01	204584902	68194967	2.30	0.10

DF = degree of freedom, SS = sum of squares, MS = mean squares, the p values <0.05 indicate that the corresponding individual and interactive effects are significant, while p values >0.05 denote that the corresponding individual and interactive effects are non-significant.

**Fig 1:**
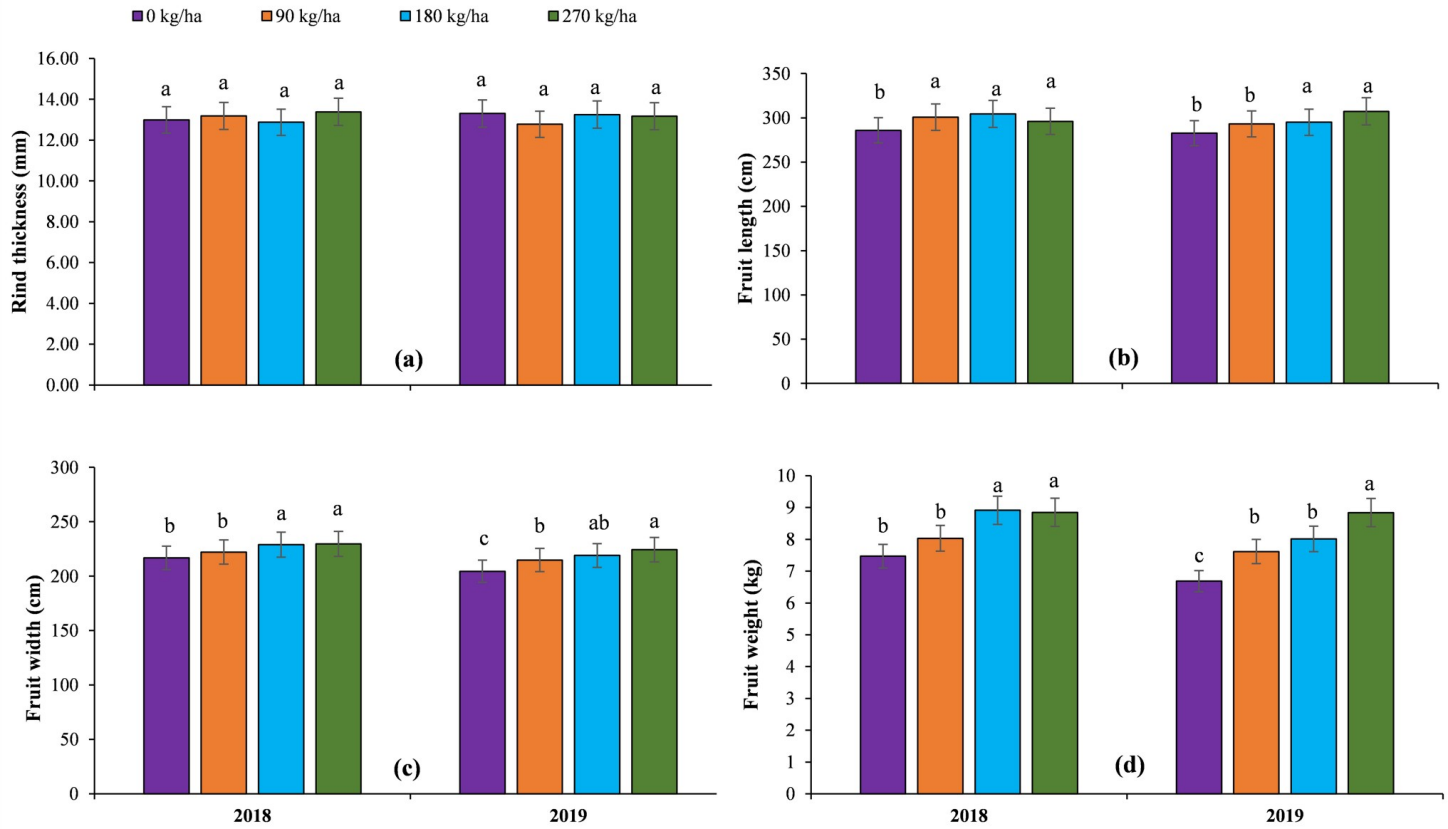
The impact of different nitrogen doses on growth attributes of grafted watermelon under field conditions.

The highest values of growth attributes such as main stem length, stem diameter and number of nodes per stem and yield were recorded for 270 kg ha^-1^ N during both years, whereas no N application observed the lowest values of these traits (Fig 1). The growth attributes were not influenced at all by B doses during both years (Table 2).

**Table 2:** The impact of different boron doses on the pomological attributes of grafted watermelon grown under climatic conditions of Çukurova region.

Boron doses	Rind Thickness (mm)	Fruit length (cm)	Fruit width (cm)	Fruit weight (kg)	Main stem length (cm)	Main stem diameter (mm)	Number of nodes	Number of branches	Yield (kg ha^-1^)
**2018**									
**0 kg ha**^**-1**^	12.60 b	285.27 b	216.60 b	7.48 b	174.34	8.91	23.06	3.26	46621 b
**2 kg ha**^**-1**^	13.61 a	307.88 a	231.95 a	9.12 a	175.44	9.03	22.44	3.29	49933 a
LSD 0.05	**1.00**	**6.69**	**4.29**	**0.40**	**NS**	**NS**	**NS**	**NS**	**2849**
**2019**									
**0 kg ha**^**-1**^	12.80 b	294.56	212.66 b	7.51 b	2.94	10.94	29.15	3.91 b	36217 b
**2 kg ha**^**-1**^	13.46 a	294.51	218.60 a	8.06 a	2.95	10.90	29.73	4.21 a	44043 a
LSD 0.05	**0.65**	**NS**	**4.56**	**0.44**	**NS**	**NS**	**NS**	**0.29**	**3937**

Means followed by similar letters within a column are statistically non-significant (p>0.05).

Regarding interactions, the highest values of all growth attributes were recorded 270 kg ha^-1^ N with both B doses during each year (Table 3).

**Table 3:** The interactive effect of different nitrogen and boron doses on the pomological attributes of grafted watermelon grown under climatic conditions of Çukurova region.

Treatments	Rind thickness (mm)	Fruit length (cm)	Fruit width (cm)	Fruit weight (kg)	Main stem length (cm)	Main stem diameter (mm)	Number of nodes	Number of branches	Yield (kg ha^-1^)
**2018**									
**N**_**1**_**B**_**1**_	12.44 cd	277.04 e	208.85 d	6.63 d	154.00 d	9.09 ab	20.25 c	3.42	32581 e
**N**_**2**_**B**_**1**_	12.40 cd	290.59 d	218.84 bc	7.81 bc	165.08 cd	8.72 b	22.00 bc	2.92	43983 c
**N**_**3**_**B**_**1**_	11.27 d	287.91 de	216.72 cd	7.35 cd	185.82 ab	8.67 b	25.09 a	3.36	54470 ab
**N**_**4**_**B**_**1**_	14.17 a	285.77 de	221.99 bc	8.12 bc	193.42 a	9.14 ab	25.08 a	3.33	55454 ab
**N**_**1**_**B**_**2**_	13.55 abc	294.82 cd	224.73 bc	8.32 b	175.00 bc	8.59 b	22.58 bc	3.33	38173 d
**N**_**2**_**B**_**2**_	13.96 ab	310.81 ab	225.55 b	8.25 b	173.08 bc	8.87 ab	23.08 ab	3.17	51787 b
**N**_**3**_**B**_**2**_	14.35 a	319.64 a	240.13 a	10.34 a	167.00 cd	9.20 ab	20.58 c	3.25	53269 ab
**N**_**4**_**B**_**2**_	12.60 bcd	306.26 bc	237.38 a	9.57 a	186.67 ab	9.46 a	23.50 ab	3.42	56505 a
**LSD 0.05**	**1.42**	**13.38**	**8.58**	**0.81**	**17.64**	**0.70**	**2.42**	**NS**	**4030**
**2019**									
**N**_**1**_**B**_**1**_	14.30 a	283.08 c	203.90 d	6.49 f	2.74 c	9.97 d	28.00 c	3.67 c	29581 d
**N**_**2**_**B**_**1**_	13.23 ab	294.14 bc	211.17 cd	7.38 de	2.85 bc	10.42 bcd	29.08 abc	4.00 abc	35213 d
**N**_**3**_**B**_**1**_	13.36 ab	297.47 abc	216.92 bc	7.58 cde	2.86 bc	11.19 abc	29.36 abc	3.73 bc	36150 cd
**N**_**4**_**B**_**1**_	12.94 b	303.81 ab	219.02 bc	8.58 ab	3.31 a	12.21 a	30.16 ab	4.25 ab	43925 bc
**N**_**1**_**B**_**2**_	12.31 b	282.33 c	205.17 d	6.88 ef	2.71 c	10.14 cd	28.67 bc	4.17 abc	29713 d
**N**_**2**_**B**_**2**_	12.32 b	292.28 bc	218.53 bc	7.85 bcd	3.02 b	10.74 bcd	29.50 abc	4.42 a	43469 bc
**N**_**3**_**B**_**2**_	13.15 ab	292.72 bc	220.91 ab	8.41 abc	2.98 bc	11.26 abc	29.75 abc	4.17 abc	50481 ab
**N**_**4**_**B**_**2**_	13.41 ab	310.73 a	229.79 a	9.10 a	3.09 ab	11.44 ab	31.00 a	4.08 abc	52513 a
**LSD 0.05**	**1.31**	**15.14**	**9.13**	**0.88**	**0.27**	**1.16**	**2.13**	**0.58**	**7946**

N_1_ = 0 kg N ha^-1^, N_2_ = 90 kg N ha^-1^, N_3_ = 180 kg N ha^-1^, N_4_ = 270 kg N ha^-1^, B_1_ = 0 kg B ha^-1^, B_2_ = 2 kg B ha^-1^, Means followed by similar letters within a column are statistically non-significant (p>0.05).

### Pomological attributes

Different N and B doses and their interaction significantly altered various pomological attributes with some exceptions (Table 1). Nitrogen doses significantly altered all pomological attributes except rind thickness during both years. Similarly, B doses also had significant effect on all pomological attributes during both years. Regarding N by B interactions, all pomological attributes were significantly affected during both years (Table 1).

**Fig 2:**
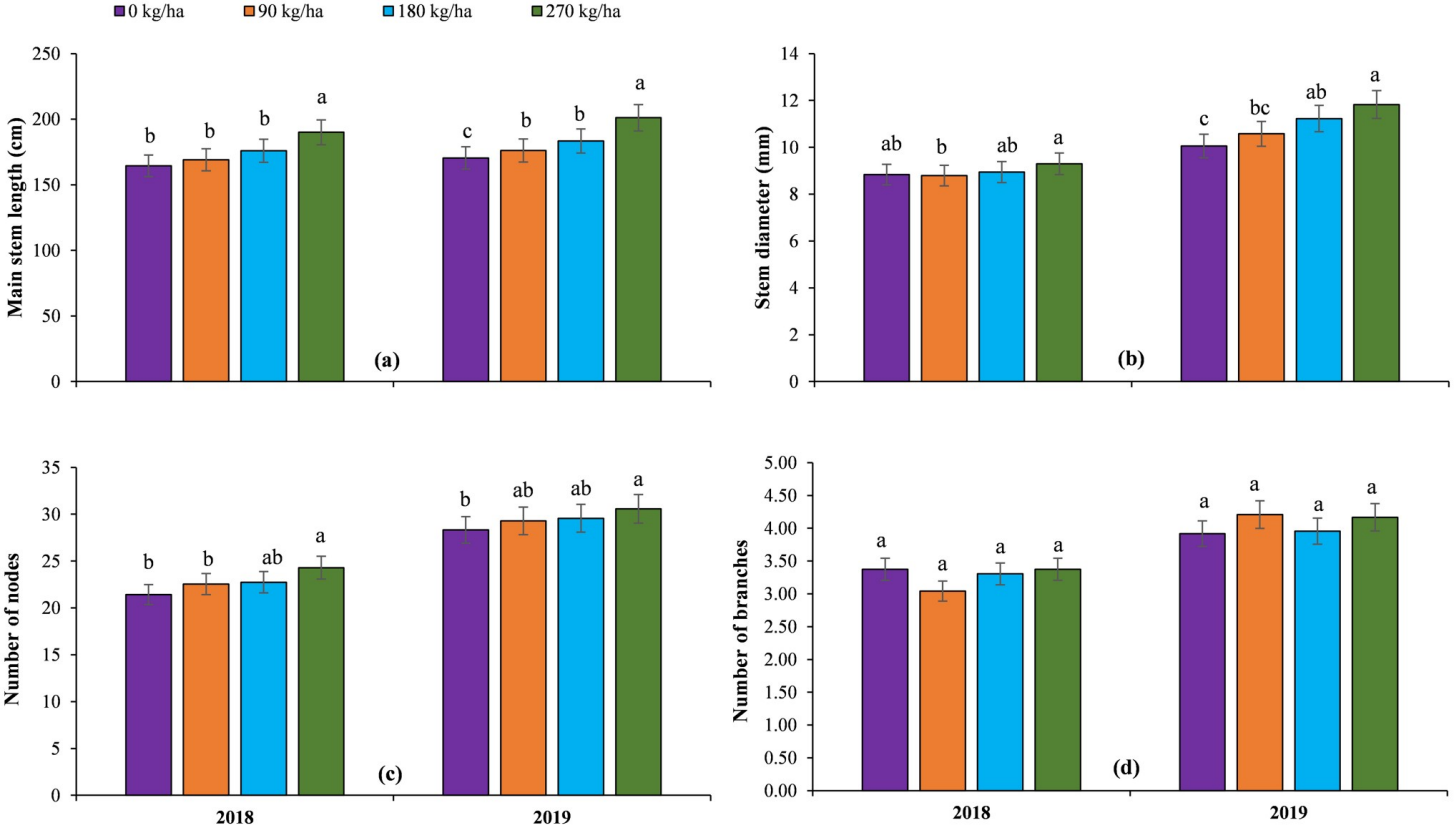
The impact of different nitrogen doses on pomological attributes of grafted watermelon under field conditions.

The highest values of pomological attributes such fruit length, width and weight were recorded for 270 kg ha^-1^ N during both years, whereas no N application observed the lowest values of these traits (Fig 2). The application of 2 kg ha^-1^ B recorded the highest values of all pomological attributes both years (Table 2).

Regarding interactions, the highest values of all pomological attributes were recorded for 270 kg ha^-1^ N with both B doses during each year (Table 3).

### Fruit quality attributes

The individual and interactive effect of N and B significantly altered fruit quality attributes during both years (Table 4). Nitrogen doses significantly altered all fruit quality attributes, including sucrose, glucose, fructose, citric acid, tartaric acid, maleic acid and ascorbic acid during both years. Similarly, different B doses had significant effect on all fruit quality attributes during both years, with some exceptions. Regarding N by B interactions, all fruit quality attributes were significantly affected during both years (Table 4).

**Table 4:** Analysis of variance for different quality traits of grafted watermelon grown under varying nitrogen and boron levels.

		2018				2019			
Sucrose									
Source	DF	SS	MS	F value	P value	SS	MS	F value	P value
**Nitrogen (N)**	3	20.68	6.89	176.66	0.0001	16.79	5.60	214.77	0.0001
**Boron (B)**	1	1.94	1.94	49.64	0.0001	4.29	4.29	164.72	0.0001
**N × B**	3	1.37	0.46	11.71	0.0001	2.22	0.74	28.39	0.0001
**Glucose**									
**Nitrogen (N)**	3	19.08	6.36	130.56	0.0001	0.23	0.08	78.78	0.0001
**Boron (B)**	1	0.03	0.03	0.52	0.4796	0.25	0.25	258.47	0.0001
**N × B**	3	0.34	0.11	2.32	0.1008	0.68	0.23	231.96	0.0001
**Fructose**									
**Nitrogen (N)**	3	0.10	0.03	4.92	0.0084	1.25	0.42	128.72	0.0001
**Boron (B)**	1	0.50	0.50	75.16	0.0001	0.00	0.00	0.60	0.4478
**N × B**	3	0.53	0.18	26.79	0.0001	0.13	0.04	13.72	0.0001
**Citric acid**									
**Nitrogen (N)**	3	792133.63	264044.54	313.11	0.0001	683016.93	227672.31	278.94	0.0001
**Boron (B)**	1	11204.11	11204.11	13.29	0.00	26318.95	26318.95	32.25	0.0001
**N × B**	3	239876.40	79958.80	94.82	0.0001	55285.59	18428.53	22.58	0.0001
**Tartaric acid**									
**Nitrogen (N)**	3	4859.90	1619.97	219.76	0.0001	1073.24	357.75	77.77	0.0001
**Boron (B)**	1	59.02	59.02	8.01	0.0093	162.84	162.84	35.40	0.0001
**N × B**	3	210.73	70.24	9.53	0.0003	595.39	198.46	43.14	0.0001
**Maleic acid**									
**Nitrogen (N)**	3	260685.56	86895.19	1.87	0.1613	1490809.88	496936.63	27.66	0.0001
**Boron (B)**	1	26628.38	26628.38	0.57	0.4562	95069.22	95069.22	5.29	0.0304
**N × B**	3	1069188.29	356396.10	7.68	0.0009	350690.65	116896.88	6.51	0.0022
**Ascorbic acid**									
**Nitrogen (N)**	3	0.52	0.17	77.44	0.0001	2.75	0.92	696.62	0.0001
**Boron (B)**	1	0.23	0.23	101.25	0.0001	0.00	0.00	0.01	0.9306
**N × B**	3	0.57	0.19	84.59	0.0001	0.75	0.25	188.50	0.0001

DF = degree of freedom, SS = sum of squares, MS = mean squares, the p values <0.05 indicate that the corresponding individual and interactive effects are significant, while p values >0.05 denote that the corresponding individual and interactive effects are non-significant.

The highest values of all fruit quality attributes except maleic acid were recorded for 270 kg ha^-1^ N during both years, whereas no N application observed the lowest values of these traits (Table 5). Similarly, 2 kg ha^-1^ B observed the highest values of fruit quality attributes during both years (Table 6).

**Table 5:** The impact of different nitrogen doses on the quality attributes of grafted watermelon grown under climatic conditions of Çukurova region.

Nitrogen doses	Sucrose	Glucose	Fructose	Citric acid	Tartaric acid	Maleic acid	Ascorbic acid
**2018**							
**0 kg ha**^**-1**^	1.06 d	1.54 d	3.50 b	378.22 d	28.81 d	2687.72 ab	0.92 d
**90 kg ha**^**-1**^	1.92 c	1.99 c	3.49 b	561.35 c	40.16 c	2841.58 a	1.04 b
**180 kg ha**^**-1**^	2.42 b	2.74 b	3.46 b	695.14 b	49.89 b	2634.28 ab	0.99 c
**270 kg ha**^**-1**^	3.28 a	3.57 a	3.61 a	798.91 a	62.28 a	2609.13 b	1.26 a
**LSD 0.05**	**0.20**	**0.22**	**0.08**	**29.96**	**2.80**	**222.32**	**0.04**
**2019**							
**0 kg ha**^**-1**^	2.74 d	1.75 b	2.99 a	454.65 d	23.64 c	2927.60 a	1.13 b
**90 kg ha**^**-1**^	3.14 c	1.91 a	2.64 b	650.05 c	32.70 b	2843.46 a	1.03 c
**180 kg ha**^**-1**^	3.99 b	1.71 c	2.47 d	755.26 b	31.79 b	2363.47 c	1.10 b
**270 kg ha**^**-1**^	4.60 a	1.69 c	2.57 c	847.55 a	39.99 a	2672.93 b	1.76 a
**LSD 0.05**	**0.16**	**0.03**	**0.05**	**29.48**	**2.21**	**138.31**	**0.03**

Means followed by similar letters within a column are statistically non-significant (p>0.05).

**Table 6:** The impact of different boron doses on the quality attributes of grafted watermelon grown under climatic conditions of Çukurova region.

Boron doses	Sucrose	Glucose	Fructose	Citric acid	Tartaric acid	Maleic acid	Ascorbic acid
**2018**							
**0 kg ha**^**-1**^	1.92 b	2.49	3.64 a	627.11 a	46.64 a	2722.02	1.13 a
**2 kg ha**^**-1**^	2.41 a	2.43	3.39 b	589.69 b	43.92 b	2664.33	0.97 b
**LSD 0.05**	**0.14**	**NS**	**0.05**	**21.19**	**1.98**	**NS**	**0.03**
**2019**							
**0 kg ha**^**-1**^	3.25 b	1.68 b	2.68	648.20 b	29.77 b	2756.37 a	1.25
**2 kg ha**^**-1**^	3.98 a	1.86 a	2.66	705.56 a	34.28 a	2647.36 b	1.25
**LSD 0.05**	**0.11**	**0.02**	**NS**	**20.84**	**1.56**	**97.80**	**NS**

Means followed by similar letters within a column are statistically non-significant (p>0.05).

Regarding interactions, the highest values of all fruit quality attributes were recorded for 270 kg ha^-1^ N with both B doses during each study year (Table 7).

**Table 7:** The impact of nitrogen by boron doses interaction on the fruit quality attributes of grafted watermelon grown under climatic conditions of Çukurova region.

Treatments	Sucrose	Glucose	Fructose	Citric acid	Tartaric acid	Maleic acid	Ascorbic acid
**2018**							
**N**_**1**_**B**_**1**_	0.57 f	1.42 e	3.75 a	249.17 e	32.33 f	2536.37 bc	0.78 e
**N**_**2**_**B**_**1**_	1.74 e	1.98 cd	3.66 ab	652.71 c	42.04 e	3175.91 a	1.22 b
**N**_**3**_**B**_**1**_	2.47 c	2.85 b	3.37 de	756.61 b	52.89 c	2598.72 bc	1.09 c
**N**_**4**_**B**_**1**_	2.91 b	3.71 a	3.77 a	849.96 a	59.31 b	2577.08 bc	1.45 a
**N**_**1**_**B**_**2**_	1.55 e	1.66 de	3.25 e	507.26 d	25.29 g	2839.07 b	1.05 c
**N**_**2**_**B**_**2**_	2.09 d	2.01 c	3.32 de	469.98 d	38.28 e	2507.24 c	0.86 d
**N**_**3**_**B**_**2**_	2.36 cd	2.63 b	3.55 bc	633.66 c	46.89 d	2669.83 bc	0.89 d
**N**_**4**_**B**_**2**_	3.64 a	3.44 a	3.44 cd	747.87 b	65.24 a	2641.17 bc	1.06 c
**LSD 0.05**	**0.28**	**0.32**	**0.11**	**42.38**	**3.96**	**314.41**	**0.06**
**2019**							
**N**_**1**_**B**_**1**_	2.48 f	1.64 e	3.01 a	354.38 e	26.21 d	3016.41 a	1.01 e
**N**_**2**_**B**_**1**_	3.15 de	1.74 d	2.75 b	640.17 c	32.46 c	2996.14 a	1.02 e
**N**_**3**_**B**_**1**_	3.34 d	1.49 g	2.45 e	757.27 b	22.67 b	2461.85 d	1.35 c
**N**_**4**_**B**_**1**_	4.03 c	1.85 c	2.50 de	840.98 a	37.75 e	2551.09 cd	1.64 b
**N**_**1**_**B**_**2**_	2.99 e	1.86 c	2.98 a	554.93 d	21.07 e	2838.79 ab	1.24 d
**N**_**2**_**B**_**2**_	3.13 de	2.08 a	2.53 d	659.93 c	32.94 c	2690.78 bc	1.05 e
**N**_**3**_**B**_**2**_	4.64 b	1.94 b	2.49 de	753.24 b	40.90 a	2265.10 e	0.84 f
**N**_**4**_**B**_**2**_	5.17 a	1.54 f	2.64 c	854.12 a	42.22 a	2794.76 b	1.88 a
**LSD 0.05**	**0.23**	**0.04**	**0.08**	**41.69**	**3.13**	**195.62**	**0.05**

N_1_ = 0 kg N ha^-1^, N_2_ = 90 kg N ha^-1^, N_3_ = 180 kg N ha^-1^, N_4_ = 270 kg N ha^-1^, B_1_ = 0 kg B ha^-1^, B_2_ = 2 kg B ha^-1^, Means followed by similar letters within a column are statistically non-significant (p>0.05).

## Discussion

Individual and interactive effects of N and B altered growth, yield, pomological and fruit quality attributes. As hypothesized, increasing doses of N and B improved growth, pomological and fruit quality attributes. The highest values of growth, pomological and fruit quality attributes were recorded for the highest N and B doses used in the current study. These can be owed to the continuous availability of both nutrients throughout the growing season, which resulted in proper functioning, metabolism and nutrition. Collectively all metabolic processes improved growth, pomological and fruit quality attributes of grafted watermelon in the current study.

Stem length or plant height is an important character related to plant productivity [34]. Main stem length was increased with increasing N doses. This could be attributed to the ability of N treatments to enhance plant growth via promoting cellular division and nutrients’ uptake from soils [35]. Chlorophyll is an essential biomolecule for photon absorption, transmission, transportation and photosynthetic rate (Pn) in leaves [36]. Lawlor et al. [37] reported that increasing N fertilizer can restore the chlorophyll in plants. Thus, improved growth attributes are owed to higher chlorophyll synthesis with higher N dose in the current study.

Plants require N in large quantity for optimum growth and development. Nitrogen is necessary for various metabolic processes of crop plants. Chlorophyll synthesis and photosynthesis are directly influenced by N [29, 38–41]. Plant growth is poor under low N availability as it is part of amino acids, nucleic acid, proteins, chlorophyll and hormones [42]. Nonetheless, photosynthesis, flowering and fruit development are positively influenced by optimum N availability resulting in higher crop yields [29, 43, 44]. Improved growth attributes are owed to these processes in the current study.

Boron is involved in numerous physiological processes of plants [20, 45, 46]. Principally, cell wall structural integration, and linkage of B with pectic polysaccharide rhamnogalacturonan regulate porosity and tensile strength of the cell wall [19]. However, limitation or excess of B adversely affect plant growth [21–24]. The obvious response of B-deficiency in several crops is inhibition of root growth because of reduced cell division [47]. Moreover, long-term deficient B condition provokes lipid peroxidation and reduces the activity of antioxidant enzymes [20, 23].

Composition of cereal grains is significantly altered by N [48], whereas sugar concentrations and conversion of simple sugars and complex carbohydrates is also regulated by N availability [49]. Free amino acid concentrations of cereal grain is significantly affected by N supply [50–52]. Concentrations of sugars in tubers may be increased by 100% in potatoes under N-deficiency [53]. Similarly, non-availability of N results in lower concentration of reducing sugars [54]. Increased N fertilizer has also been shown to cause a rise in free amino acid concentrations [55]. The improvement in fruit quality attributes, particularly of sugars is directly linked with N availability. Since both B doses resulted in similar fruit quality attributes with both B doses, it is concluded that N is the main determinant of fruit quality attributes rather than B.
